# Network Pharmacology-Based Prediction of Active Ingredients and Mechanisms of *Lamiophlomis rotata* (Benth.) Kudo Against Rheumatoid Arthritis

**DOI:** 10.3389/fphar.2019.01435

**Published:** 2019-11-27

**Authors:** Yunbin Jiang, Mei Zhong, Fei Long, Rongping Yang, Yanfei Zhang, Tonghua Liu

**Affiliations:** ^1^College of Pharmaceutical Sciences and Chinese Medicine, Southwest University, Chongqing, China; ^2^College of Pharmacy, Chengdu University of Traditional Chinese Medicine, Chengdu, China; ^3^Institute of Tibetan Medicine, Tibetan Traditional Medical College, Lhasa, China; ^4^Key Laboratory on Health Cultivation of the Ministry of Education, Beijing University of Chinese Medicine, Beijing, China

**Keywords:** *Lamiophlomis rotata*, rheumatoid arthritis, network pharmacology, active ingredient, mechanism, luteolin, PI3K-Akt signaling pathway

## Abstract

**Background:** *Lamiophlomis rotata* (LR) showed favorable clinical effect and safety on rheumatoid arthritis (RA), but its active ingredients and mechanisms against RA remain unknown. The aim of this work was to explore the active ingredients and mechanisms of LR against RA by network pharmacology.

**Methods:** Compounds from LR were identified using literature retrieval and screened by absorption, distribution, metabolism, excretion, and toxicity (ADMET) evaluation. Genes related to the selected compounds or RA were identified using public databases, and the overlapping genes between compounds and RA target genes were identified using Venn diagram. Then, the interactions network between compounds and overlapping genes was constructed, visualized, and analyzed by Cytoscape software. Finally, pathway enrichment analysis of overlapping genes was carried out on Database for Annotation, Visualization, and Integrated Discovery (DAVID) platform.

**Results:** A total of 148 compounds in LR were identified, and ADMET screen results indicated that 67 compounds exhibited good potential as active ingredients. A total of 90 compounds-related genes and 1,871 RA-related genes were identified using public databases, and 48 overlapping genes between them were identified. Cytoscape results suggested that the active ingredients and target genes of LR against RA consisted of 23 compounds and 48 genes, and luteolin and AKT1 were the uppermost active ingredient and hub gene, respectively. DAVID results exhibited that the mechanisms of LR against RA were related to 34 signaling pathways, and the key mechanism of LR against RA might be to induce apoptosis of synovial cells by inactivating PI3K-Akt signaling pathway.

**Conclusion:** The active ingredients and mechanisms of LR against RA were firstly investigated using network pharmacology. This work provides scientific evidence to support the clinical effect of LR on RA, and a research basis for further expounding the active ingredients and mechanisms of LR against RA.

## Introduction

Rheumatoid arthritis (RA), a chronic autoimmune disease, can cause cartilage and bone damage as well as disability. RA is characterized by joint inflammation, but is more like a syndrome that consists of extra-articular manifestations, such as rheumatoid nodules, pulmonary involvement or vasculitis, and systemic comorbidities (Smolen et al., 2016). RA can present at any age, affects about 1% of the population, and carries a huge emotional and financial burden for both the individual and society (McInnes and Schett, 2017). Because inflammation is the main driving factor to cause clinical symptoms, joint damage, disability, and comorbidity in RA patients, anti-inflammation is a key therapeutic strategy (Smolen et al., 2007). At present, the anti-RA drugs include disease-modifying antirheumatic drugs and non-steroidal anti-inflammatory drugs in western country (Smolen et al., 2016). However, traditional Chinese medicine (TCM) plays a vital complementary role in treating RA in China (Zhang et al., 2010). For the past few years, TCM has been increasingly important strategy for treatment of RA in China due to its good therapeutic effect and low toxic side effects.

Chinese Pharmacopoeia shows that *Lamiophlomis rotata* (Benth.) Kudo (LR) can be used to treat RA, and LR patent medicines (Duyiwei capsule or tablet) are legally allowed to trade in China. It was reported that LR showed favorable clinical effect and safety on RA (Ye et al., 2007), and a meta-analysis indicated that LR was effective and safe in treating bleeding, pain, and inflammation (Wang et al., 2008). In addition, animals experiment indicated that LR could significantly inhibit the formation of primary and secondary arthritis in rats (Wang et al., 2013). At present, the active ingredients and mechanisms of LR against RA has not been reported. Therefore, the studies on active ingredients and mechanisms of LR against RA should be strengthened to provide scientific evidence to support its clinical application in treating RA.

Network pharmacology, a systematic analytical method, can analyze the interaction network of multiple factors such as drugs, protein target, diseases, and genes (Hopkins, 2007). Network pharmacology can decipher the mechanism of drugs action with a holistic perspective, which emphasizes the paradigm shift from “one target, one drug” to “network target, multicomponent therapeutics” (Hopkins, 2008). The characteristic is also shared by TCM, and the holistic theory has long been central to TCM treatments of various diseases (Li et al., 2014). Therefore, network pharmacology is a very advantageous technology to explore TCM-related issues. At present, network pharmacology has been widely used to investigate the active ingredients and mechanisms of TCM against various diseases (Tang et al., 2015; Chen et al., 2018).

**Figure 1:**
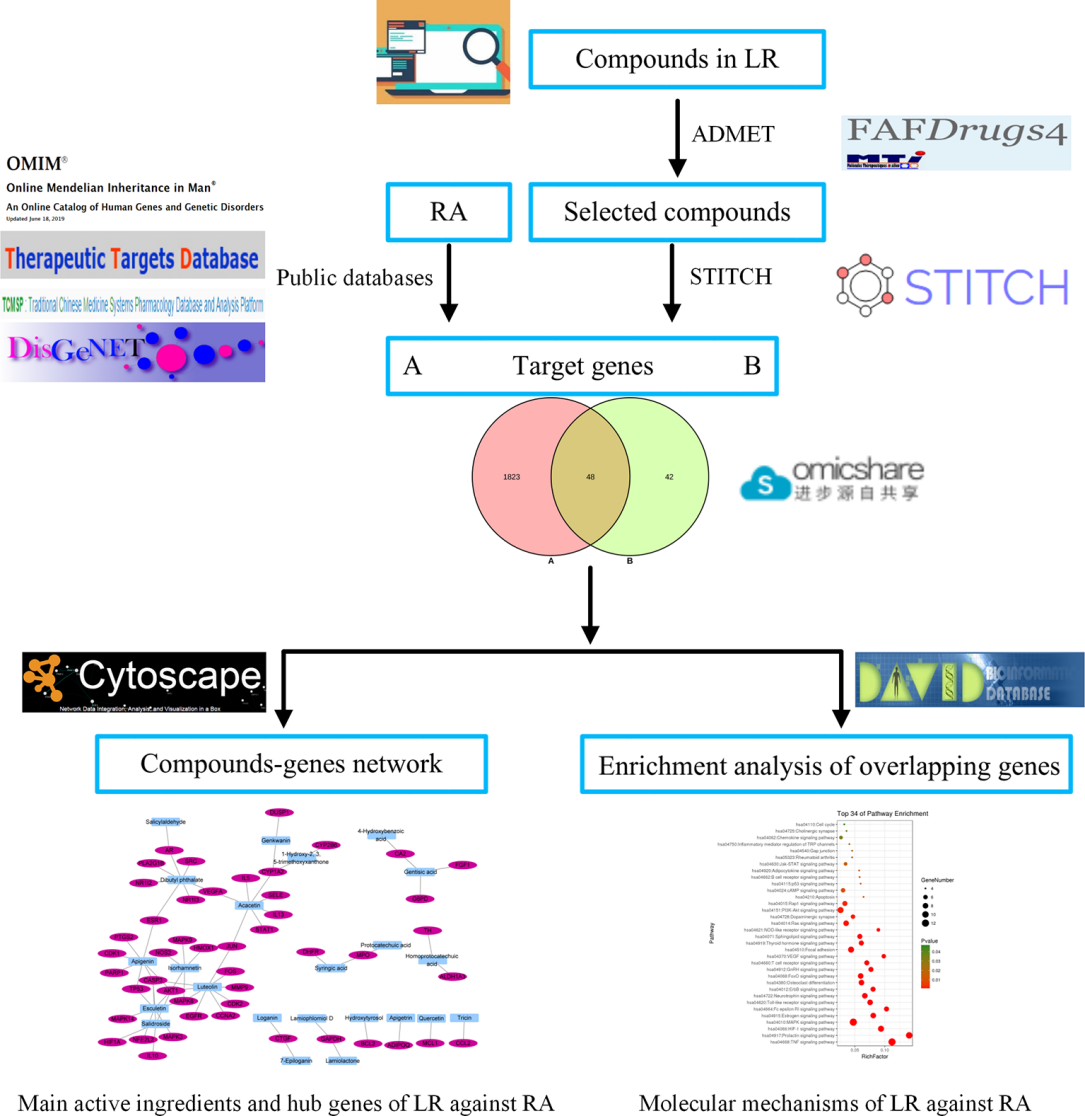
Workflow of network pharmacology analysis.

In this work, network pharmacology was used to investigate the active ingredients and mechanisms of LR against RA. First, compounds from LR were identified using literature retrieval, and were screened by absorption, distribution, metabolism, excretion, and toxicity (ADMET) evaluation. Then, genes related to selected compounds or RA were identified using public databases, and the overlapping genes between compounds and RA target genes were identified. Third, the key active ingredients and hub genes of LR against RA were identified by analyzing the interactions between compounds and overlapping genes. Finally, pathway enrichment analysis of overlapping genes was carried out to explore the molecular mechanisms of LR against RA. The workflow is shown in **Figure 1**.

## Materials and Methods

### Compounds Database Construction and ADMET Evaluation

The information of compounds from LR were collected by retrieving literatures in CNKI (http://www.cnki.net/), WANFANG DATA (http://www.wanfangdata.com.cn/), Baidu Xueshu (http://xueshu.baidu.com/), Web of Science and Google Scholar, and the SMILES and molecular formulas of compounds were identified using SciFinder (https://scifinder.cas.org/), PubChem (https://pubchem.ncbi.nlm.nih.gov/), or ChemSpider (http://www.chemspider.com/) with the aid of compounds names or structure. Then, compounds were screened by applying ADMET criteria of FAFDrugs4 (http://fafdrugs4.mti.univ-paris-diderot.fr/) (Miteva et al., 2006) with the aid of SMILES, and the “PhysChem Filters” of FAFDrugs4 was set as “*Drug-Like Soft.*” Compounds were selected out as potential active ingredients when the result of ADMET evaluation was “Accepted.”

### Target Genes Linked to Selected Compounds or RA

Based on SMILES, target genes of the identified compounds were predicted using STITCH (http://stitch.embl.de/) (Szklarczyk et al., 2016) with the “*Homo sapiens*” setting. To get more credible target genes of each compound, compound with the highest “Tanimoto score,” usually 1.000 (match *via* InChIKey), was used to predict the genes of target compound, and the target genes were screened by setting “minimum required interaction score” as “high confidence (0.700)” during performing STITCH prediction (Lee et al., 2018).

RA-related target genes were identified by retrieving public databases including Online Mendelian Inheritance in Man (OMIM, https://omim.org/), Therapeutic Target Database (TTD, http://bidd.nus.edu.sg/group/cjttd/) (Li et al., 2018), Traditional Chinese Medicine Systems Pharmacology Database and Analysis Platform (TCMSP, http://lsp.nwu.edu.cn/tcmsp.php) (Ru et al., 2014), and DisGeNET (http://www.disgenet.org/). The overlapping genes between compounds and RA target genes were identified and visualized by Venn diagram, plotted using the OmicShare tools, a free online platform for data analysis (www.omicshare.com/tools).

### Network Construction of Interactions Between Compounds and Overlapping Genes

The interactions between compounds and overlapping genes were obtained based on the results of STITCH prediction, and the network of the interactions was constructed, visualized, and analyzed by Cytoscape ver. 3.7.1 (https://cytoscape.org/). Nodes in network indicate compounds and genes, and edges suggest interactions between compounds and genes (Lee et al., 2018). The key active ingredients and hub genes of LR against RA were selected out by setting “Degree value” of compounds or genes, identified by analyzing topological structure of network. Degree value of compounds or genes represents the edges numbers of compounds or genes in network. The bigger degree value of compounds or genes are, the more important compounds or genes are for the therapeutic effect of LR on RA.

### Pathway Enrichment Analysis of Overlapping Genes

Kyoto Encyclopedia of Genes and Genomes (KEGG) pathway enrichment analysis of overlapping genes was carried out on Database for Annotation, Visualization, and Integrated Discovery ver. 6.8 (https://david.ncifcrf.gov/) with the “*Homo sapiens*” setting. The results of KEGG pathway enrichment were used to decipher the potential molecular mechanisms of LR against RA. Bubble chart of interested KEGG pathways was plotted by the OmicShare tools.

## Results

### Potential Active Ingredients From LR

A total of 148 compounds in LR were identified by literatures retrieval, and the names, molecular formulas of these compounds are listed in **Supplementary Table S1**. The ADMET screen results of 148 compounds showed that the results of 67 compounds were “Accepted,” indicating that the 67 compounds exhibited good potential as active ingredients. These compounds are listed in **Table 1**.

**Table 1:** A list of the final selected 67 compounds in LR for network analysis based on ADMET screen.

No.	Compound	No.	Compound
1	(−)-α-terpineol-8-O-β-D-glucopyranoside	35	gentisic acid
2	(+)-α-terpineol-8-O-β-D-glucopyranoside	36	hexanoic acid
3	(2Z)-2,6-dimethyl-6-hydroxyocta-2,7-dienyl-O-β-D-glucopyranoside	37	homoprotocatechuic acid
4	(E)-4-hydroxyhex-2-enoic acid	38	hydroxytyrosol
5	(Z)-3-hexenyl glucopyranoside	39	icariside H1
6	1-hydroxy-2,3,5-trimethoxyxanthone	40	isololiolide
7	2,4,5-trihydroxycinnamic acid	41	isorhamnetin
8	3,4-dihydroxybenzaldehyde	42	lamiolactone
9	3β-hydroxy-5α,6α-epoxy-7-megastigmen-9-one	43	lamiophlomiol A
10	4’-(p-carbonylphenyl)-luteolin	44	lamiophlomiol B
11	4-hydroxybenzoic acid	45	lamiophlomiol C
12	5-hydroxyloganin	46	lamiophlomiol D
13	7,8-dehydropenstemonoside	47	lamiophlomiol E
14	7,8-dehydropenstemoside	48	lamiophlomiol F
15	7-dehydroxyzaluzioside	49	lamiophlomis alkali
16	7-deoxyloganic acid	50	loganin
17	7-deoxyloganin	51	loliolide
18	7-epiloganin	52	luteolin
19	8-deoxyshanzhiside	53	n-butyl-β-D-fructofuranoside
20	8-epi-7-deoxyloganin	54	n-butyl-β-D-fructopyranoside
21	8-epideoxyloganic acid	55	notohamosin B
22	acacetin	56	penstemoside
23	apigenin	57	phlorigidoside C
24	apigetrin	58	protocatechuic acid
25	caffeic acid	59	quercetin
26	cedrol	60	rhexifoline
27	chlorogenic acid	61	salicylaldehyde
28	chlorotuberoside	62	salidroside
29	cyclohexylglycine	63	salviifoside A
30	dibutyl phthalate	64	shanzhiside methyl ester
31	dodecanoic acid	65	syringic acid
32	esculetin	66	tricin
33	eugenyl-O-β-D-glucopyranoside	67	vanillyl-O-β-D-glucopyranoside
34	genkwanin		

ADMET, absorption, distribution, metabolism, excretion and toxicity; LR, Lamiophlomis rotate.

### Target Genes Linked to the 67 Compounds or RA

**Figure 2:**
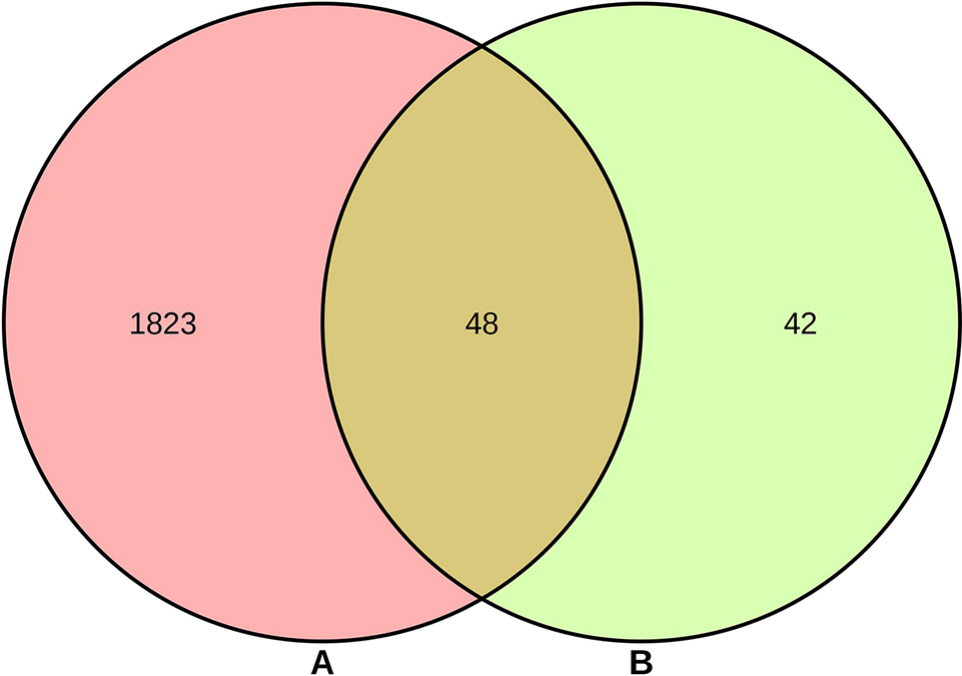
Overlapping genes between 1,871 rheumatoid arthritis (RA)-related genes **(A)** and 90 compounds-related genes **(B)**.

As shown in **Supplementary Table S2**, a total of 90 genes related to 25 compounds from abovementioned 67 compounds were identified using STITCH prediction, and no genes linked to another 42 compounds were identified based on STITCH prediction. As listed in **Supplementary Table S3**, a total of 1,871 RA-related genes were identified by retrieving OMIM, TTD, TCMSP, and DisGeNET databases. The results of Venn diagram (**Figure 2**) suggested 48 overlapping genes were identified by matching 90 compounds-related genes with 1,871 RA-related genes.

### Key Active Ingredients and Hub Genes of LR Against RA

**Figure 3:**
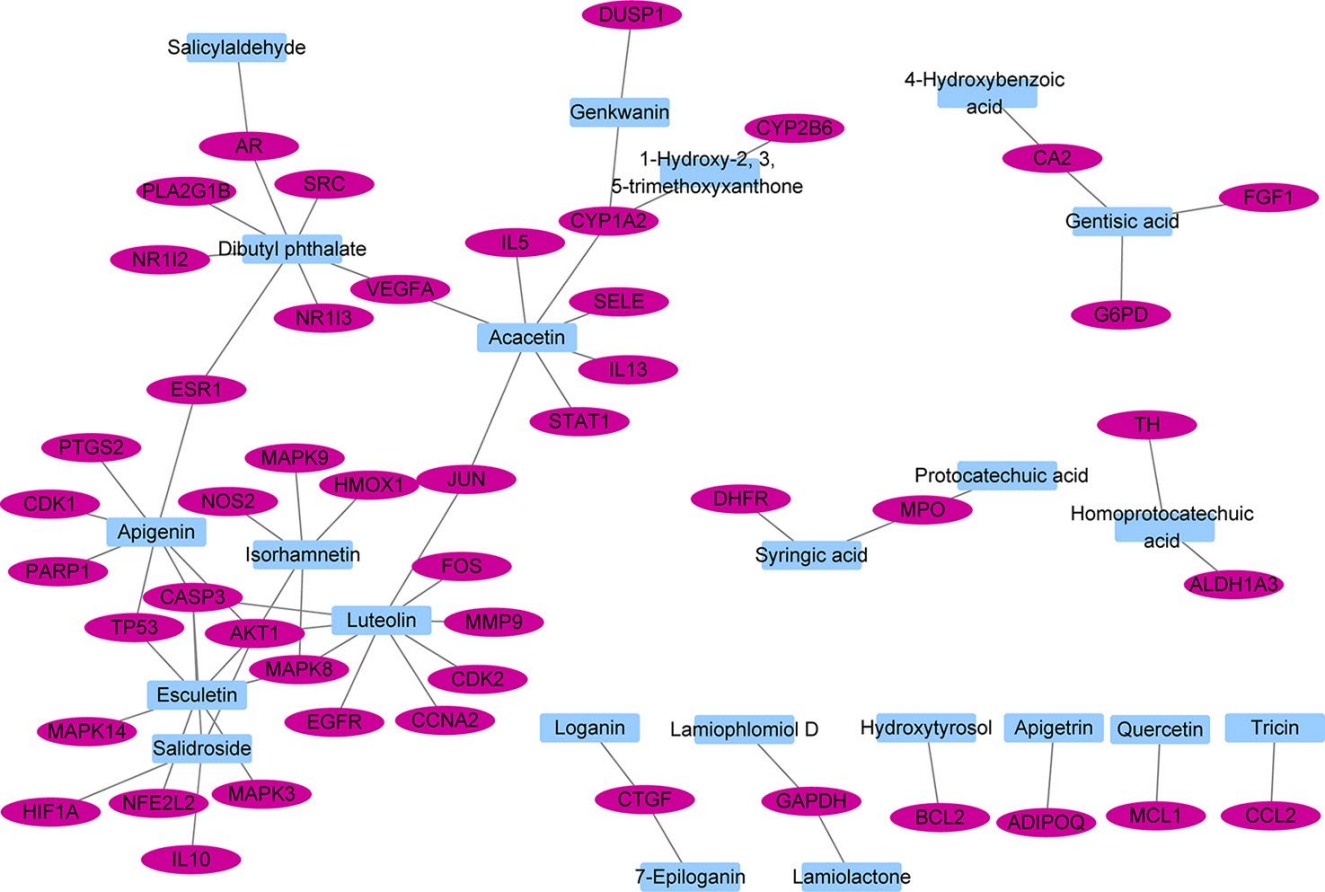
Network with 71 nodes and 68 edges linking 23 compounds in *Lamiophlomis rotata* and 48 target genes related to rheumatoid arthritis.

**Figure 4:**
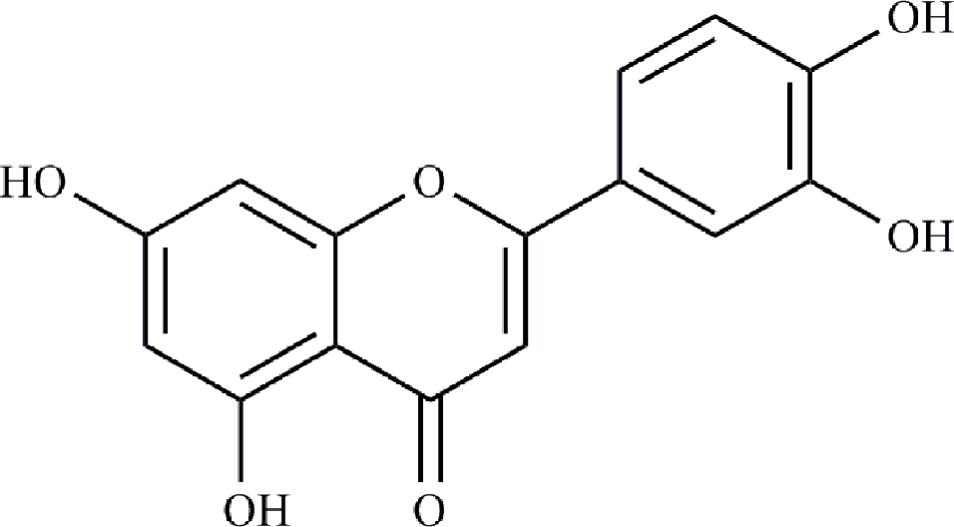
Chemical structure of luteolin.

The interactions between 48 overlapping genes and compounds were identified based on the results of STITCH prediction, and 23 compounds were finally identified. The interactions between 48 overlapping genes and 23 compounds are listed in **Table 2**, and were visualized by network, which includes with 71 nodes and 68 edges (**Figure 3**). The results suggested that the therapeutic effect of LR on RA was directly related to the 23 compounds and 48 genes. The 23 compounds were categorized as nine flavonoids (luteolin, apigenin, acacetin, isorhamnetin, genkwanin, 1-hydroxy-2,3,5-trimethoxyxanthone, quercetin, tricin, and apigetrin), five phenolic acids (gentisic acid, syringic acid, homoprotocatechuic acid, protocatechuic acid, and 4-hydroxybenzoic acid), four iridoids (loganin, 7-epiloganin, lamiophlomiol D, and lamiolactone), two volatile oil (dibutyl phthalate and salicylaldehyde), one coumarin (esculetin), one phenylethanoid glycoside (salidroside), and one polyphenol (hydroxytyrosol). Based on the degree value of each compound or gene (**Table 3**), it was very easy to distinguish the contribution difference of 23 compounds and 48 genes to LR against RA. Luteolin (**Figure 4**), connected to nine genes, was considered as the uppermost active ingredient of LR against RA. AKT1, connected to five compounds, was considered as the hub gene of LR against RA.

**Table 2:** A list of the interactions between 23 compounds in LR and 48 target genes related to RA.

No.	Compound	Gene	No.	Compound	Gene
1	1-hydroxy-2,3,5-trimethoxyxanthone	CYP1A2	35	genkwanin	CYP1A2
2	1-hydroxy-2,3,5-trimethoxyxanthone	CYP2B6	36	gentisic acid	FGF1
3	4-hydroxybenzoic acid	CA2	37	gentisic acid	G6PD
4	7-epiloganin	CTGF	38	gentisic acid	CA2
5	acacetin	IL5	39	homoprotocatechuic acid	TH
6	acacetin	SELE	40	homoprotocatechuic acid	ALDH1A3
7	acacetin	VEGFA	41	hydroxytyrosol	BCL2
8	acacetin	IL13	42	isorhamnetin	NOS2
9	acacetin	STAT1	43	isorhamnetin	MAPK9
10	acacetin	CYP1A2	44	isorhamnetin	HMOX1
11	acacetin	JUN	45	isorhamnetin	MAPK8
12	apigenin	CDK1	46	isorhamnetin	AKT1
13	apigenin	PTGS2	47	lamiolactone	GAPDH
14	apigenin	ESR1	48	lamiophlomiol D	GAPDH
15	apigenin	CASP3	49	loganin	CTGF
16	apigenin	PARP1	50	luteolin	CCNA2
17	apigenin	TP53	51	luteolin	CASP3
18	apigenin	AKT1	52	luteolin	EGFR
19	apigetrin	ADIPOQ	53	luteolin	FOS
20	dibutyl phthalate	NR1I3	54	luteolin	MAPK8
21	dibutyl phthalate	ESR1	55	luteolin	CDK2
22	dibutyl phthalate	VEGFA	56	luteolin	AKT1
23	dibutyl phthalate	PLA2G1B	57	luteolin	JUN
24	dibutyl phthalate	SRC	58	luteolin	MMP9
25	dibutyl phthalate	NR1I2	59	protocatechuic acid	MPO
26	dibutyl phthalate	AR	60	quercetin	MCL1
27	esculetin	NFE2L2	61	salicylaldehyde	AR
28	esculetin	MAPK14	62	salidroside	CASP3
29	esculetin	CASP3	63	salidroside	IL10
30	esculetin	MAPK8	64	salidroside	HIF1A
31	esculetin	MAPK3	65	salidroside	AKT1
32	esculetin	TP53	66	syringic acid	DHFR
33	esculetin	AKT1	67	syringic acid	MPO
34	genkwanin	DUSP1	68	tricin	CCL2

LR, Lamiophlomis rotate; RA, rheumatoid arthritis.

**Table 3:** Degree value of 23 compounds and 48 target genes in network.

No.	Compound	Value	No.	Gene	Value	No.	Gene	Value
1	luteolin	9	1	AKT1	5	25	TH	1
2	apigenin	7	2	CASP3	4	26	PTGS2	1
3	acacetin	7	3	CYP1A2	3	27	DUSP1	1
4	esculetin	7	4	MAPK8	3	28	CCNA2	1
5	dibutyl phthalate	7	5	MPO	2	29	IL13	1
6	isorhamnetin	5	6	ESR1	2	30	MCL1	1
7	salidroside	4	7	VEGFA	2	31	CCL2	1
8	gentisic acid	3	8	CTGF	2	32	STAT1	1
9	syringic acid	2	9	CA2	2	33	PARP1	1
10	homoprotocatechuic acid	2	10	TP53	2	34	HMOX1	1
11	genkwanin	2	11	GAPDH	2	35	IL10	1
12	1-hydroxy-2,3,5-trimethoxyxanthone	2	12	JUN	2	36	PLA2G1B	1
13	hydroxytyrosol	1	13	AR	2	37	EGFR	1
14	protocatechuic acid	1	14	NOS2	1	38	FOS	1
15	quercetin	1	15	CDK1	1	39	G6PD	1
16	tricin	1	16	BCL2	1	40	MAPK3	1
17	loganin	1	17	IL5	1	41	HIF1A	1
18	7-epiloganin	1	18	FGF1	1	42	SRC	1
19	4-hydroxybenzoic acid	1	19	MAPK9	1	43	CDK2	1
20	lamiophlomiol D	1	20	SELE	1	44	ALDH1A3	1
21	lamiolactone	1	21	DHFR	1	45	CYP2B6	1
22	salicylaldehyde	1	22	NFE2L2	1	46	NR1I2	1
23	apigetrin	1	23	NR1I3	1	47	MMP9	1
			24	MAPK14	1	48	ADIPOQ	1

### Potential Molecular Pathways of LR Against RA

**Figure 5:**
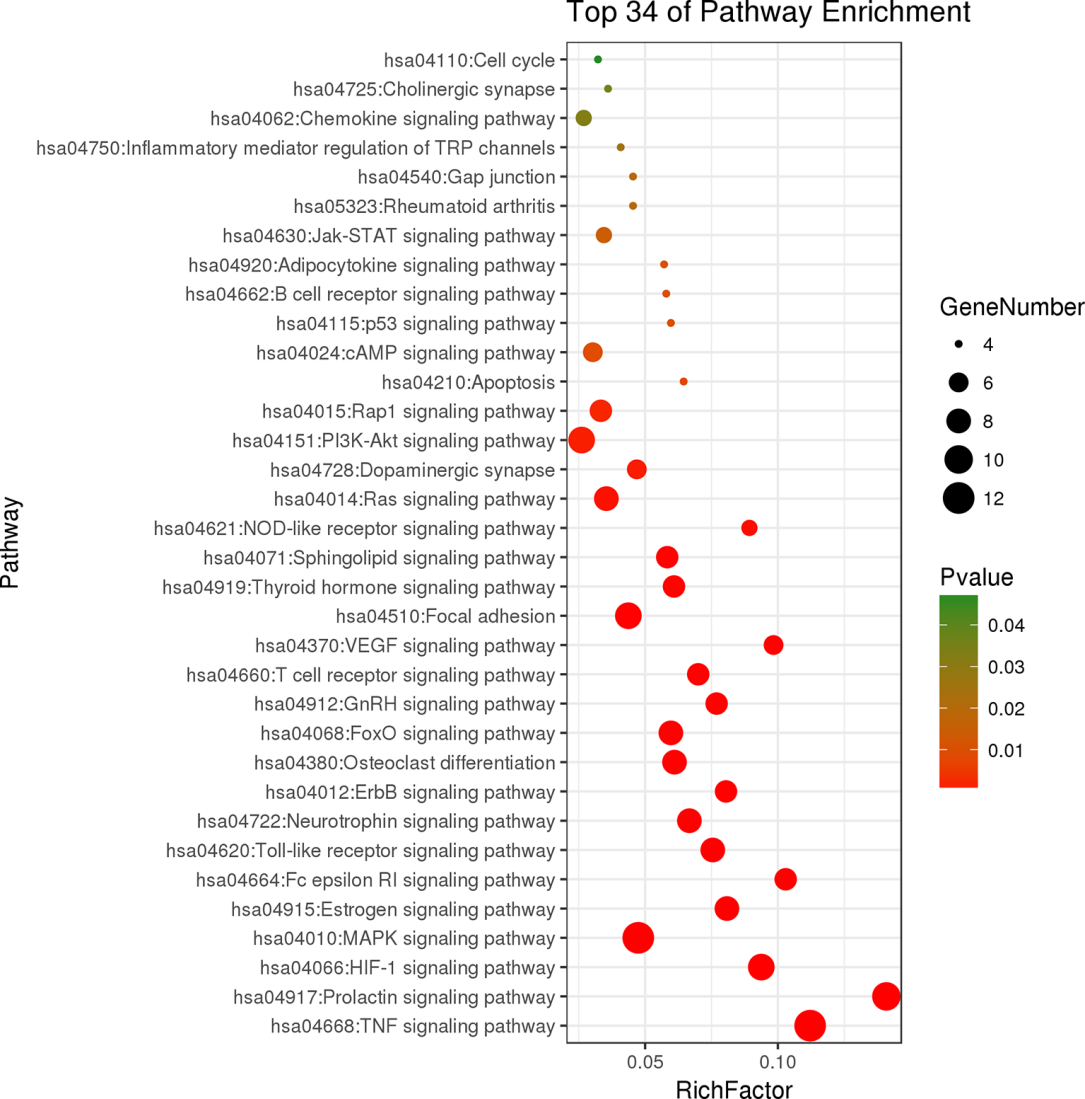
Bubble chart of 34 signaling pathways related to occurrence and development of rheumatoid arthritis.

The results of KEGG pathway enrichment analysis indicated that 48 overlapping genes were significantly enriched in 74 signaling pathways (*p* < 0.05). Based on the extensive literature retrieval, the 34 signaling pathways (**Figure 5**) were directly related to occurrence and development of RA, indicating that these signaling pathways might be the mechanisms of LR against RA. The detailed information of top 10 pathways is shown in **Table 4**. In addition, the hub gene AKT1 of LR against RA was directly enriched in 27 signaling pathways of the 34 signaling pathways. Coincidently, AKT1 plays a role in almost all of the 27 signaling pathways by PI3K-Akt signaling pathway, suggesting that PI3K-Akt signaling pathway might be the hub signaling pathway of LR against RA.

**Table 4:** Target genes in top 10 of pathway enrichment related to occurrence and development of RA.

Pathway ID	Term	Target genes
hsa04668	TNF signaling pathway	AKT1, FOS, CASP3, CCL2, PTGS2, MAPK14, JUN, MMP9, MAPK3, MAPK9, MAPK8, SELE
hsa04917	Prolactin signaling pathway	AKT1, FOS, MAPK14, MAPK3, TH, ESR1, MAPK9, MAPK8, STAT1, SRC
hsa04066	HIF-1 signaling pathway	AKT1, EGFR, HIF1A, HMOX1, BCL2, MAPK3, VEGFA, NOS2, GAPDH
hsa04010	MAPK signaling pathway	AKT1, EGFR, FOS, CASP3, DUSP1, MAPK14, JUN, MAPK3, TP53, MAPK9, MAPK8, FGF1
hsa04915	Estrogen signaling pathway	AKT1, EGFR, FOS, JUN, MMP9, MAPK3, ESR1, SRC
hsa04664	Fc epsilon RI signaling pathway	AKT1, IL5, MAPK14, MAPK3, MAPK9, IL13, MAPK8
hsa04620	Toll-like receptor signaling pathway	AKT1, FOS, MAPK14, JUN, MAPK3, MAPK9, MAPK8, STAT1
hsa04722	Neurotrophin signaling pathway	AKT1, MAPK14, JUN, BCL2, MAPK3, TP53, MAPK9, MAPK8
hsa04012	ErbB signaling pathway	AKT1, EGFR, JUN, MAPK3, MAPK9, MAPK8, SRC
hsa04380	Osteoclast differentiation	AKT1, FOS, MAPK14, JUN, MAPK3, MAPK9, MAPK8, STAT1

RA, rheumatoid arthritis.

## Discussion

Compounds-genes network suggested that the therapeutic effect of LR on RA was directly related to 23 compounds, including nine flavonoids, five phenolic acids, four iridoids, two volatile oil, one coumarin, one phenylethanoid glycoside, and one polyphenol. The ratio of flavonoids to 23 compounds was close to 40%, suggesting that flavonoids were more important than other kinds of compounds for the therapeutic effect of LR on RA. Based on the degree value of each compound in compounds-genes network, luteolin was considered as the uppermost active ingredient of LR against RA. It was reported that flavonoids were the key active ingredients group of LR (Lin et al., 2003), and flavonoids are used to control the quality of LR patent medicines (Duyiwei capsule or tablet) in Chinese Pharmacopoeia. Studies suggested that luteolin inhibited the proliferation and partially blocked the pathogenic function of synovial fibroblasts in RA (Hou et al., 2009; Lou et al., 2015). Meanwhile, TCMSP suggests that luteolin is related to occurrence and development of RA (Ru et al., 2014). Additionally, it was reported that the quantity of luteolin in LR was about 0.9% (Yi and Sun, 2016), and the clinical dosage of LR patent medicines is 9 g/day based on Chinese Pharmacopoeia, suggesting that the daily intake of luteolin 81 mg in clinic. Study indicated that luteolin showed obvious anti-RA effect on mice with collagen type II-induced RA at a dose of 1 mg/kg/day (Impellizzeri et al., 2013), which is far lower than the equivalent dose of luteolin in mice, suggesting that the quantity of luteolin in LR is high enough to be of pharmacological relevance.

Compounds-genes network showed that the therapeutic effect of LR on RA was directly related to 48 genes. The results of KEGG pathway enrichment analysis of 48 genes suggested that 34 signaling pathways were directly linked to occurrence and development of RA, indicating that these signaling pathways might be the mechanisms of LR against RA. The relationships of the top 10 pathways with RA were briefly discussed as follows. TNF signaling pathway: The occurrence and development of RA can be suppressed by inhibiting the overexpression of TNF-α, and antibody therapy against TNF-α can effectively reduce the arthritis and synovitis symptoms of RA patients (Matsuno et al., 2002). Prolactin signaling pathway and estrogen signaling pathway: Sex hormones such as estrogen and prolactin have long been thought to be directly related to occurrence and development of RA, and recent evidence indicated that estrogen and prolactin showed both anti- and pro-inflammatory effects in RA (Tang et al., 2017). HIF-1 signaling pathway: Clinical research exhibited that HIF-1alpha level was strongest in the sub-lining layer of RA synovium and was linked to synovium inflammation and angiogenesis in RA patients (Brouwer et al., 2009). MAPK signaling pathway: It was reported that andrographolide showed protective effects on RA through inhibiting MAPK pathways, suggesting that MAPK signaling pathway was related to occurrence and development of RA (Li et al., 2017). Fc epsilon RI signaling pathway: Report indicated that IgE, the initiation factor in Fc epsilon RI signaling pathway, may be involved in some extra-articular manifestations of RA (Meretey et al., 1982). Toll-like receptor signaling pathway: Previous reports indicated that Toll-like receptor and the signaling pathway were intensively linked to RA pathogenesis (Takagi, 2011). Neurotrophin signaling pathway: Report suggested that the level of mesencephalic astrocyte-derived neurotrophic factor was closely related to occurrence and development of RA (Ma et al., 2018). ErbB signaling pathway: It was reported that ErbB-2 was involved in occurrence and development of RA (Jiang et al., 2012). Osteoclast differentiation: Reports exhibited that activated RA synovial fibroblasts played a vital role in rheumatoid bone destruction by expressing osteoclast differentiation factor (Shigeyama et al., 2000).

Based on the degree value of each gene in compounds-genes network, AKT1 was considered as the hub gene of LR against RA. AKT1 was directly enriched in 27 signaling pathways of the abovementioned 34 signaling pathways. Coincidently, AKT1 plays a role in almost all of the 27 signaling pathways by PI3K-Akt signaling pathway, suggesting that PI3K-Akt signaling pathway might be the hub signaling pathway of LR against RA. Joint synovium is the main diseased region in RA patients, and its out-of-control proliferation to cartilage and bone causes release of inflammatory cytokines, resulting in occurrence of RA. Therefore, inducing apoptosis of synovial cells is also a feasible strategy for treating RA by preventing development of inflammation (Park et al., 2010). It was reported that PI3K-Akt signaling pathway was abnormally activated in RA synovium, resulting in the overexpression of anti-apoptotic genes such as FLIP, Bcl-2, and Mcl-1 (Harris et al., 2009). The overexpression of these anti-apoptotic genes lead to out-of-balance apoptosis of synovial cells, which induced occurrence and development of RA (Smith et al., 2010). Reports indicated that luteolin, the uppermost active ingredient of LR against RA, inhibited the proliferation of synovial fibroblasts in RA by blocking PI3K-Akt signaling pathway (Hou et al., 2009). Therefore, the key mechanism of LR against RA might be to induce apoptosis of synovial cells by inactivating PI3K-Akt signaling pathway.

## Conclusion

The active ingredients and mechanisms of LR against RA were firstly investigated using network pharmacology. The findings of this work suggested that the active ingredients and target genes of LR against RA consisted of 23 compounds and 48 genes, and luteolin and AKT1 were the uppermost active ingredient and hub gene of LR against RA, respectively. The mechanisms of LR against RA were related to 34 signaling pathways, and the key mechanism of LR against RA might be to induce apoptosis of synovial cells by inactivating PI3K-Akt signaling pathway. This work provides scientific evidence to support the clinical effect of LR on RA, and a research basis for further expounding the active ingredients and mechanisms of LR against RA.

## Data Availability Statement

The raw data supporting the conclusions of this manuscript will be made available by the authors, without undue reservation, to any qualified researcher.

## Author Contributions

YJ, YZ, and TL conceived and designed this work, and wrote and revised the whole manuscript. MZ collected the data. YJ, MZ, FL, and RY analyzed the data.

## Funding

This work was supported by the Regional Collaborative Innovation Center Project of Tibetan Medicine (No. 2018XTCX045).

## Conflict of Interest

The authors declare that the research was conducted in the absence of any commercial or financial relationships that could be construed as a potential conflict of interest.
